# Rationing cancer treatment: a qualitative study of perceptions of legitimate limit-setting

**DOI:** 10.1186/s12913-018-3137-3

**Published:** 2018-05-09

**Authors:** Eli Feiring, Hege Wang

**Affiliations:** 1 Department of Health Management and Health Economics, University of Oslo, PO Box 1089, Blindern, 0317 Oslo, Norway; 2 Department of Guidelines and Professional Development, Norwegian Directorate of Health, PO Box 7000, St Olavs plass, 0130 Oslo, Norway

**Keywords:** Rationing, Priority-setting, Legitimacy, High-cost, Cancer drugs, Clinicians, Thematic analysis, Norway

## Abstract

**Background:**

Governments are facing tough choices about whether to fund new, promising but highly expensive drugs within the public healthcare system. Decisions that some drugs are not sufficiently beneficial relative to their cost to merit public funding are often contentious. The importance of making decisions that stakeholders can understand and accept as legitimate is increasingly recognized and is commonly understood to be a crucial component for stakeholder support and successful implementation. Yet, little is known about clinicians’ perceptions of legitimate limit-setting. This study aimed to examine oncologists’ perceptions of the legitimacy of governmental decisions to deny patients access to new cancer drugs because effectiveness and cost-effectiveness of the drugs has not been demonstrated.

**Methods:**

Semi-structured in-depth interviews with 12 Norwegian oncologists were carried out. Data were interpreted with the use of theory driven thematic analysis. The analytical framework of Accountability for reasonableness aided data gathering and interpretation.

**Results:**

The participants endorsed the ideal of explicit criteria-based priority setting. Yet, when confronted with actual rationing decisions, they were far more equivocal. They advocated for increased access to drugs and were not always prepared to accept rationing of drugs they felt would benefit their patient. Distrust in the Norwegian centralised drug review process was found and different rationales were identified: i) Lack of engagement with the process, ii) Disagreement with the use of rationing criteria, iii) Lack of transparency and lack of dispute resolution procedures. Concerns about the wider implications of rationing decisions were reported. Most importantly, these related to negative impact on patient-doctor relationship of micro-level rationing and to inequities in drug availability resulting from privatisation of high-cost cancer treatment.

**Conclusions:**

Drawing on the analytical framework, we conclude that perceptions of legitimacy regarding rationing of high-cost drugs include procedural fairness. However, notions of substantive justice also seem to be important for accepting reasons given for decisions. Regulatory legitimacy may further warrant a more sophisticated theoretical account of second-order beliefs about the justifiability of rationing new technologies. These findings indicate a need for a broader concept of legitimacy than is commonly used in the literature on healthcare prioritisation.

## Background

All societies must make decisions about what healthcare interventions to provide to their population. In most high-income countries, including Norway, an aging population, rising consumer expectations and technological innovation have contributed to increased demand for healthcare resources. Setting priorities is necessary in order to ensure sustainability of the healthcare system and difficult decisions on resource allocation are required.

Drug funding policies have a major influence on macro-level priority-setting in a given country. Pharmacotherapies play an important role in modern healthcare and new drugs are potentially very expensive. High-cost drug therapies used to treat cancer are among several factors contributing to the fact that the cost of delivering high-quality equitable cancer care is a challenge to national budgets even in affluent countries [1–4]. At the same time, effectiveness of the drugs is sometimes limited or uncertain or may benefit only a subgroup of the patients, and cost-effectiveness of the drugs is consequently poor. This situation has led to a debate over rationing high-cost cancer drugs, i.e. limiting the possibilities to an optimal satisfaction of healthcare needs.

Cancer is one of the leading causes of morbidity and mortality worldwide and a disease from which people seek protection at “any” cost [5]. Affordability of end-of-life treatment is a subject of particular controversy [6]. Decisions that some cancer drugs are not sufficiently beneficial relative to their cost to merit public funding tend to be contentious. Thus, rationing decisions take place against a background of substantial political discomfort. Decisions about which cancer drugs to fund within a public healthcare system affect different stakeholders in different ways, and governmental decision-makers recognize the importance of perceived legitimacy of their funding processes [3]. Theoretical frameworks for legitimate and fair priority setting, such as Daniels and Sabin’s Accountability for Reasonableness [7], emphasize the importance of rigor of the process and transparency of the data and rationale by which decisions are made. Empirical studies have identified weaknesses of current allocation processes, such as concerns about transparency of funding processes and limited opportunities for stakeholder participation in the processes [3], and have underscored how well-designed processes can help the public to understand how decisions were made, and recognize the process and methods used, and eventually accept the outcome that follows as justified [8]. Many countries (s. a. the UK, Canada, Australia, New Zealand, and Norway) have implemented centralised drug review processes to assist in making drug coverage decisions [2]. These processes contributes to systems of explicit rationing, where allocation decisions are made according to agreed criteria and where patients are both aware that they are denied treatment and have had the reasons for this denial explained to them [9, 10].

While other studies have examined attitudes applied by decision-makers [3, 11–13], empirical work on the experiences of explicit rationing at the micro level is sparse. A recent study researched patients’ reactions to rationing [14]. Some studies have explored rationing decisions in general practice [15, 16], and others have examined barriers to drug access and their effect on specialist clinical practice [17–19]. Still, little attention has been given to clinicians’ views about justifiability and legitimacy of limited drug access (but see [20, 21] for health professionals‘views on rationing albeit not in cancer).

To our knowledge, there are hardly any qualitative study of oncologists’ perceptions of the legitimacy of restricting access to new and expensive cancer drugs available. A recent study of the role of oncologists in rationing cancer care in Germany (using quantitative and qualitative data), found that job satisfaction was negatively affected by cost considerations and negotiations in 72% of the respondents [22]. Further, the respondents were concerned about the quality of care as a result of implicit rationing, and the authors highlighted the need for the development of explicit criteria which are ethically and legally more justifiable. The study was, however, conducted in a setting different from ours and where rationing is not done explicitly and denied by politicians.

We wanted to examine how oncologists experience governmental decisions to deny patients access to new cancer drugs within the public healthcare system because effectiveness and cost-effectiveness of the drugs has not been demonstrated, and their perceptions of legitimate limit-setting. For the purpose of this study, we hypothesised that oncologists’ perceptions of the justifiability of public agencies’ regulative decisions are imperative to their acceptance of the role assigned to oncologists of steward of medical resources.

This paper reports the findings from the case study investigation, detailing experiences of oncologists. Before outlining the methodology, we briefly describe the Norwegian setting.

## Methods

### Study design

This study was designed as a theoretically informed qualitative case study. A case study makes it possible to study a single unit in detail, linking general knowledge to empirical knowledge of the more specific mechanisms in an individual case [23]. We wanted theory-before-research to assist design research questions, guide the selection of relevant data, aid in defining an appropriate description and interpretation of data, and eventually help move beyond specific insights from the single case we set out to study [24]. We used thematic analysis to identify, analyse and report patterns within data [25]. We did not aim to explore a full range of themes grounded in the data itself (saturation), but to give a detailed analysis of some theoretically driven aspects of the data.

### Theoretical framework

There is a range of different value judgments inherent in healthcare allocation decisions and there is often persistent disagreement on ethical principles to guide decisions. Empirical studies have further illustrated how rationing decisions affect a range of stakeholders, all of whom make different claims for resources and refer to different underlying allocation principles [3]. This seems to be an argument for making decisions which is seen to be in accordance with acceptable procedural criteria. Therefore, notions of procedural justice and legitimacy are increasingly used to justify emphasis on priority-setting processes and outcomes, rather than principles.

Legitimacy may be interpreted purely descriptively to refer to actual beliefs and social acceptance or normatively, to refer to justifications of authority and obligation [26]. However, some authors have argued for a concept of legitimacy that combines descriptive and normative elements, and Daniels and Sabins’ novel framework Accountability of reasonableness [7] should be understood in this vein. The framework is ethics-based and may be used to examining the fairness and legitimacy of priority setting processes.

According to Accountability for reasonableness, fair allocation of resources requires that health care decision-makers are accountable for the reasonableness of their regulative behaviour. This means that decisions about setting limits must rest on evidence, reasons and principles that all stakeholders consider relevant (relevance condition). Further, decisions must be publicly accessible and there must be opportunities for revision and improvement of policies in light of new evidence or arguments (publicity and revision conditions). The decision-making process should be regulated to ensure that these conditions are met (regulative condition). Daniels and Sabin underscore the importance of seeking agreement on how, rather than what decisions are made. It is important that even stakeholders that disagree with the final outcome are able to accept it as legitimate and fair, given the process. So, for the process to be legitimate, it also has to be acceptable by those it seeks to govern.

On this conception of legitimacy, different claims are invoked both about the goals which the decision-makers are pursuing such as justice claims, and claims about performance, such as the extent to which they operate in accordance with relevance norms, scientific norms, or norms of transparency and openness [27]. The process itself is a process of legitimation.

In this paper, we utilised the framework for descriptive rather than normative purposes. We wanted theory to assist design and research questions and guide data-selection, description and interpretation. We assumed that notions of reasonableness, such as relevance, transparency, revisability, and regulative authority, are important to the individual oncologists’ assessment of regulations that imply restrictions of clinical autonomy for societal purpose. We further assumed that “legitimacy enhances compliance and compliance enhances legitimacy” and that the oncologists’ second order beliefs about legitimate decision-making, i.e. their beliefs about the justifiability of rationing high-cost cancer drugs, are imperative for their compliance. Overall, we assumed that the process would gain in legitimacy if it is complied with because the objective delegated to the centralised drug review process, namely explicit principled rationing, can only be promoted if clinicians comply.

### Setting

Norway is characterised by high per capita income and an egalitarian ideological orientation. Healthcare is universal and tax-financed, and 8,9% of GDP is spent on health [28]. While primary healthcare and social services in Norway are organised at the municipality level, an independent local administrative level, specialised healthcare is subject to national governance. Specialised healthcare is administered by four regional health enterprises.

The main health policy goal is to offer good quality healthcare equally accessible to all. There are at least three different concerns of equity at play. First, healthcare should be distributed according to need. Second, there should be no discrimination on the basis of personal and social characteristics, such as social status groups, genders, ethnicity, and age, place of residence, diagnosis, or cause of illness. Third, health authorities should aim to reduce unfair social inequalities in health. Further, solidarity is an important underlying value and is expressed in the health system’s single tier design that aims to share the best standard of care possible with all members of society. Thus, the patients’ ability to pay does not enter the decision-making process concerning use of expensive technology in hospitals. Recently this model has, however, been challenged by an increase in the number of Norwegians holding private health insurance. The role of insurance in health care financing is however negligible [28]. The number of private hospitals is very small, but there is reported an increase in patients seeking drug infusion in private clinics.

Norway has a long history of systematic discussions and implementation of explicit priority-setting where politically defined guidelines and processes regulate prioritising [29]. National allocation-criteria are both established and broadly accepted and are understood as to enhance the likelihood that resources are allocated fairly and transparently. Access to specialist healthcare and reimbursements schemes for pharmaceuticals is regulated by law. According to the Norwegian Patient Rights Act of 1999, Norwegians have a legal right to necessary hospital treatment, which is defined according to two effectiveness criteria: expected benefit from healthcare, and cost-effectiveness. A third criterion, severity, should also be assessed to determine for how long the patient may wait for healthcare to be given. No explicit cost-effectiveness thresholds are defined.

Following an international trend, Norwegian health governance emphasised the facilitation of prioritisation processes and deliberation between stakeholders to a larger degree during the 1990s [30]. Developments in evidence-based decision-making further led to technical processes that rely on formal, economic evaluation of interventions. Different semi-independent advisory bodies were established to provide evidence-based assessments of difficult prioritisation issues and to create transparency and legitimacy around recommendations, such as the Norwegian Knowledge Centre for Health Services and the Norwegian Council for Quality Improvement and Priority Setting.

Recently, several white papers concluded that there were great variations in practices for introduction, assessment and decision-making on technology in Norway and that formal health technology assessments have not been used routinely to inform decisions [31–33]. A “National system for the evaluation and introduction of new health technologies within the specialist health service” was introduced in 2015 to secure patient safety and equal access to new cost-effective technology, obtain coordination, cooperation and transparency and established a centralised technology review process in Norway. Proposals for the assessment of a new technology to be introduced are to be handled by the Ordering Forum and may then require a national health technology assessment provided by the Knowledge Centre. The Decision Forum, consisting of the CEOs of the regional health enterprises, reviews and provides mandatory “guidance” on drugs and other technologies. Three priority setting criteria, severity, efficacy and cost-effectiveness, in addition to neutrality in relation to diagnosis, age, and the size of the patient group, should guide the decisions. Prices are negotiated with the pharmaceutical companies and the real prices are sometimes undisclosed. To date, 25 cancer drugs have been reviewed for 40 indications, of which 18 were not approved.

### Data: Participants

We used a purposeful (non-representative) sampling to identify and select participants in this study. Twelve clinical oncologists (breast and/or prostate cancer specialists; males and females) working at large and semi-large hospitals in the four regional health enterprises were strategically selected and interviewed in 2015. The participants were invited because they were regarded as experienced and knowledgeable clinicians. The sample was partly identified ex ante according to the position and experience the participants held, and partly by chain-referral, where the initial set of participants was supplied with oncologists nominated by those already interviewed. One potential participant did not answer the invitation.

A theoretically driven approach was used to aid data gathering and interpretation. Thus, we aimed to give detailed analysis of some theoretically driven aspects of the data rather than to explore the full range of themes grounded in the data itself (saturation).

### Data collection: Interviews

Data was collected by means of qualitative in-depth expert interviews. The interviews were semi-structured and were based on an interview guide. Interviews investigated participants’ experiences of dealing with restrictions of access to new cancer drugs and their perceptions of legitimacy of priority-setting and rationing. Open-ended responses were allowed to elicit personal experiences and perspectives and to uncover as much information as possible. Interviews lasted between 30 and 60 min.

All interviews were conducted in Norwegian by the second author, audio-taped and transcribed verbatim. Each participant was given the opportunity to review the relevant transcript to identify any corrections. After coding and for the purpose of presenting the data in an international context, the first author translated the most relevant parts into English.

### Data analysis

Data was interpreted within a theoretically driven thematic analysis framework [25]. We used the six-step analysis strategy suggested by this approach. Both investigators participated in the coding and the interpretation. First, data was read through to get a sense of the overall content (step 1). Then we identified content referring to categories that were informed by our theoretical framework (explicit/implicit priority setting; priority setting criteria and process (representation, transparency, appeals mechanisms); implications of priority setting) and text units with statements were entered into tables (step 2 and 3). We continuously compared text units and themes between transcripts to ensure consistency and comprehensiveness (step 4). Finally, units were merged to themes referring to notions of legitimate limit-setting as discussed in the theoretical framework (step 5). Step 6 refers to producing the report.

## Results

Our findings are reported below, organised on the basis of themes that resulted from the analysis of the data and informed by the theoretical framework: i) Commitment to the ideal of explicit criteria-based priority setting, ii) Concern for the present patient, iii) Lack of trust in the centralised rationing process, iv) Concern for the wider implications of rationing decisions. Quotes from participants are included for illustration.

### Commitment to the ideal of explicit priority setting

The participants all endorsed the ideal of explicit criteria-based priority setting that is grounding the Norwegian individual legal right to necessary healthcare (see Setting). Characteristically, they said: “We have to prioritise, of course we have to”. They further reflected upon alternative usages of resources. The need to prioritise between healthcare and other policy areas was highlighted by some. One said:

*“It is clear that we would prefer inexhaustible resources for our patients and could offer any treatment. I mean it sincerely. But I also understand that our services form a part of a bigger picture, including traffic matters, kindergartens etc. I am strong supporter of prioritisation”.*

Others pointed out how resources alternatively could be spent within the public healthcare system. The following statement was typical:

*“As an oncologist, I can’t selfishly say that this treatment is to be introduced when the cost is two million (Norwegian kroner) per life year gained. How could the money otherwise be spent in health care? Just look at the elderly, there is a good answer. And certainly, in spite of increased focus, drug abuse and psychiatry, chronic diseases in general”.*

### Concern for the present patient

While some of the participants did not experience rationing as a challenge in their daily practice, others advocated for increased access to new and promising cancer drugs. They were not always prepared to accept rationing of drugs they felt would benefit their patient. These participants told about efforts to circumvent rationing decisions, most notably in the form of enrolling the patient on a clinical trial if they were eligible or informing the patient about private care.

The role of the doctor as patient advocate was highlighted and a concern for the present patient was evident in the data. The role was, however, considered at odds with their role as gatekeepers. The limitations imposed did not allow the participants the discretion they thought they needed to make the best treatment decisions for their patients. One of the participants said:

*“As a doctor, I can’t relate to every single directive. I have to treat the patient I have in front of me in the best possible way”.*

Another pointed out how emotional distressing micro-level rationing decisions are:

*“It is clear that we must prioritise, but when you're sitting there with the patient, then it isn’t so easy. I feel that I don’t want to relate to it”.*

Some expressed great frustration because they were not able to offer the patient treatment they thought Norway as a rich country should be willing to finance. The following statements illustrate this concern:

*“I am used to answer my patients when they ask: Yes, you are getting the best treatment that is available in the world. That I can’t say anymore”.*

*“The authorities accept that patients receive worse treatment than the academic community believes is optimal”.*

One of the participants concluded:

*“We feel like we are the last ones to adopt new medicines in Europe”.*

### Lack of trust in the centralised rationing process

As described (under Setting), Norway has implemented a centralised technology review process. The Decision Forum, consisting of the CEOs of the regional health enterprises, reviews and provides mandatory “guidance” on drugs and other technologies. In this study, distrust in the centralised drug review procedure was reported. One commonly used statement was: “*I feel that decisions are made in a way that we cannot relate to”*. Different rationales for this distrust were identified and concerned both notions of relevance, publicity and revisability of rationing decisions: i) Lack of engagement in the process, ii) Disagreement with use of rationing criteria; iii) Lack of transparency and lack of dispute resolution procedures.

#### Lack of engagement in the process

The participants in this study believed that the narrow representation of members in Decision Forum was a barrier to clinicians’ engagement in the relevant priority setting processes. They argued that the clinical perspective was missing in decision-making. One said:

*“I believe it is important to bring in medical specialists in decision-making. The level of professional knowledge varies widely, even in the Decision Forum, consisting of CEOs without any specific subject matter requirements”.*

Another reported:

*“The Decision Forum does not seek contact with the (medical) professionals. I think they should. Then one could increasingly distinguish between what is essential and what is less important”.*

Also, the participants were worried about the pool of reasons that would be aired and eventually considered in the meetings of Decision Forum. In particular, it was pointed out that economic reasoning was prevalent in the meetings and they were concerned about how the logic of health economics was dominating discussions. The two following statements from different participants were typical:

*“The (medical) professionals have been overrun by the health economists, and this undermines the (clinical) guidelines completely”.*

*“The Decision Forum participants have too much financial responsibility for their enterprises, focusing too much on economic considerations. Decisions are based more on economics than on (treatment) effect; they see almost only the money. They see the deficit coming. Should have more people who work with patients, not just people with money in their eyes. Should have been a different composition - for example, some from the different medical specialties”.*

#### Disagreement with use of rationing criteria

The participants emphasised the relevance and importance of two of the criteria used in the decision-making processes: Expected benefit from healthcare and severity. As two of the participants said:

*“I agree that it (the condition) must be severe for the individual and possibly also for the society in general (…). We agree on the treatment effect criterion - if there is no effect, it has nothing to do here”.*

*“Treatment effect is of course essential. Severity is also important – there is a difference between a matter of life and death, and living with a chronic illness”.*

However, a strong disagreement with the use of the cost-effectiveness criterion and the weight that considerations of costs had in decision-making was found in our material. Further, the participants questioned the relevance of valuing statistical or abstract life, using an estimated average cost per QALY (quality adjusted life years), and disregarding prospects of increased life expectancy of real patients. Some also argued that financing of end-of-life drugs requires a different approach. One of the participants said:

*“Then there is this: What is cost-effective? QALY might not be the best measure we have”.*

Another told about the discussions among colleagues about Decision Forum’s use of the cost-effectiveness criterion:

*“The decisions depend on the cost-effectiveness (criterion). There are plenty of calculations that we've talked about. And we who treat young and old people who are either seriously ill or dying, people with short life expectancy, we are often not listened to”.*

#### Lack of transparency and lack of dispute resolution procedures

Lack of transparency regarding the decision-making process was an important concern to the participants. They were especially worried about rationing decisions on drugs in cases where prices were not publicly disclosed. As one of the participants said

*“But with the secret negotiations, then I think that maybe they (Decision Forum) have a little to go on. I like it best when things are open and transparent”.*

Other were even more critical and expressed how they thought the secrecy of drug prices undermined the trustworthiness of the decision-making process. One pointed out how secret rebates on prices make decisions on cost-effectiveness difficult to assess:

*“It [secret prices] seems corrupt, I think. Maybe it's not so much cheaper, we do not know. Maybe it is exactly the same price. I think the prices should be public”.*

Further, concerns about the lack of a formal appeals mechanism were raised by several of the participants. The following statement was typical:

*“The decisions are perceived as absolute, which is challenging. It should have been possible to appeal or use it (the drugs) for some special patients”.*

### Concern for the wider implications of rationing decisions

Many participants reported that patients would be angry or disappointed when told about rationing decisions. The participants worried that discussions about rationing would have negative impact on the patient-doctor relationship because these discussions with patients were stressful and would often result in questions about trust. Two of the statements illustrate this concern:

*“Patients are very angry and get irritated, typically they can’t be bothered to pay for this (…). There is great frustration amongst the patients, which I have no trouble understanding”.*

*“Often they (the patients) say: Can I trust you? I know I’m not getting the best treatment”.*

The participants reflected upon whether or not to mention the possibility of new drugs available but not publicly funded. Some of these participants found the situation ethically challenging. One said:

*“I think it is a very painful situation to be in. In our department, we have to handle the fact that medications that are available in the private sector are not available from the public health service. The question is whether to provide information about this, or not at all. We have concluded that we must inform about this, but I think it is difficult. I know many patients will try everything possible, in economic terms, to be able to pay for this themselves. It can cost an enormous amount of money for very little treatment. I think it's heart breaking”.*

Another said:

*“Our ethical problem is this: If there are drugs that we can’t use, which we know could have had effects in the patient, are we obliged to inform the patient about this? If a patient who obviously can’t afford to pay for this, do we have a duty to inform or should we leave it?”.*

Many of the participants expressed the worry that rationing of high-cost drugs would stimulate privatisation of cancer treatment. They feared for the consequences for the public healthcare system and raised concern for unequal treatment between patients that can afford private healthcare if they are denied treatment within the public system and those that cannot:

*“We see that there is a class-divided healthcare system emerging, which many of us don’t appreciate”.*

As another explained:

*“It is a class-divided society. Those who have money will be offered treatment those who don’t have money don’t get”.*

## Discussion

As far as we are aware, this is the first qualitative study of oncologists’ views on the legitimacy of restrictions on access to new and expensive cancer drugs in a setting where national criteria-based priority setting is implemented and where a centralised drug review system is in place (but see [22]). This section considers key findings from the study.

The participants all endorsed the ideal of explicit criteria-based priority setting. It was emphasised that escalating healthcare costs make it necessary to make priority-setting decisions and that new expensive drugs represents a considerable challenge regarding public spending. Yet at the same time, an overarching concern for the present patient was expressed and the participants did not always seem to be prepared to accept rationing of cancer drugs that they felt could benefit the patient. Specifically, the decisions made by the centralised review body were not always regarded as authoritative. The participants questioned the rationales given for how and why limits are being set and different factors that contributed to this distrust were identified. These concerned both notions of relevance, publicity and revisability of rationing decisions. Three sub-themes were important: Lack of engagement in the process, disagreement with the use of rationing criteria and lack of transparency and dispute resolution procedures. Also, a concern for the wider implications for the public healthcare system of rationing high-cost cancer drugs was evident in the data. Two factors were important to the participants. First, patients’ diminishing trust in doctor’s advice was highlighted. Second, worries about a resulting inequity between patients that can afford private treatment and those who cannot, were expressed.

The Accountability for reasonableness framework underscores how giving reasons is a way to achieve acceptance and compliance from stakeholders who have diverse perspectives on the limit-setting decision under discussion [7]. The reasons offered must be ones that all can accept as relevant and appropriate, and the decisions and their rationales must be publicly accessible and revisable. Empirical studies have found that evidence and decisions are valued for their legitimising potential and increase in clinical support [10]. In an analysis of centralised review processes, it was concluded that the transparency, rigor, and robustness of the processes were critically important for them to be effective decision-support tool [2]. Thus, it seems to be imperative for perceived legitimacy of rationing decisions among health professionals that they recognize the value judgments underlying decision-making and that they think of the decision making process as fair. This assessment is a question both about how the process is organised and who is involved and about the normative and factual reasoning that guides decision-making.

In this study, the composition of Decision Forum was perceived as a barrier to the validity and acceptance of the rationing decisions. The participants thought that the clinical view was insufficiently taken into consideration in the decision-making processes and they wanted broader representation of different interests in the Forum. The participants did not feel engaged with the processes. This may be a major reason for not accepting limits, as found in a previous study of impact of priority setting decisions on practice of oncologists [17].

Previous studies have reported a distinction between different stakeholders’ views on the reasonableness of different criteria. According to two recent literature reviews on medicines funding decisions [3, 11], patients, patients’ advocates and clinicians generally emphasise timely access to drugs, the need for equity between different patient groups, and the need to “rescue” severely ill patients. Health authorities, on the other hand, give greater importance to the economic impact of the decisions. This research accords with previous findings. We found that the participants were critical towards Decision Forum’s use of rationing criteria. The participants expressed moral intuitions that are not always easily reconciled with societal perspectives on priority setting, which include a limit for the use of very high-cost, marginal-benefit cancer drugs near the end of life because of opportunity costs.

At least three different underlying ethical controversies are at play. The debate about how to balance considerations of cost-effectiveness against other important considerations in health priority setting, such as severity, is complex and difficult. First, cost-effectiveness (that the benefits are obtained at a reasonable cost comparable with other typically funded treatments and at a reasonable cost per QALY) can sometimes conflict with considerations of need and some think that treatment for severely ill cancer patient justifies deviances from an otherwise accepted cost-effectiveness ratio. Second, urgency may be seen to justify abandoning reasonable standards of cost-effectiveness and last chance cancer therapies may provide health benefits to those with urgent needs [34]. Third, some would argue that treatment benefits have grater value in terminal care and that “hope” for a chance of the best possible outcome is an important value for cancer patient and their families [11].These controversies should not be overlooked and indicate why different stakeholder views should be included in decision-making.

Lack of transparency regarding the decision-making and concerns about the lack of formal dispute resolution procedures were found to be another important barriers for the perceived legitimacy of the rationing decisions in this study. Specifically, the participants worried about estimates of effects and costs. This worry is not surprising given what is known from studies on introduction of cancer drugs. Even though assessment of value for money of new drugs is an important part in decision-making, studies indicate how new cancer drugs are often introduced with relatively great uncertainty about their effects [35]. Trials may be small and included patients may differ in many ways from those treated in clinical practice, and follow-up times may be short. From a clinical viewpoint, there may be indications that the effect of a drug is underestimated in the proportion of patients with good clinical benefit. Clinicians may reasonably insist that its “real” value is difficult to determine before the drug is studied in clinical practice.

Confidentiality of drug prices is becoming more common. There is no long tradition of confidential prices in Norway and there is considerable political resistance to undisclosed rebates based on the list price for the drug. A recent price comparison study of cancer drugs found that high income countries pay vastly different prices and drugs can be four times more costly in some countries than in others [4]. Some of the participants expressed that it was difficult to trust rationing decisions when they were unable to assess whether or not the price negotiated was reasonable. The findings from this study indicate that reasoning behind decisions was not available outside Decision Forum and that this lack of publicity may hinder oncologist understanding the scientific bases for decisions.

Concern for the wider implications of rationing decisions for the public healthcare system was discussed by many of the participants. The impact on doctor-patient relationship was a concern. Previous studies have concluded that limited drug funding and access has significant negative impact on oncologists’ practice [17, 18, 22]. Also, some of the participants experienced an unresolved ethical issue about whether to inform patients about drugs they did not think the patient would be able to pay for. Because they would protect patients from added concern about how to privately finance the drugs, they would not always mention the possibility of new drugs available. This well-intended but paternalistic inclination runs against the principle of respect for personal autonomy and obligation to provide patients with information that may be relevant to a treatment decision [36], and may add to distrust in doctor-patient relationship.

Many of the participants were concerned that inequities in drug availability would have negative impact on cancer outcomes for those unable to pay privately. “Ability-to-pay-rationing”, i.e. that poorer patients who cannot afford private treatment will be denied drugs, while better off patients will obtain them, was an unacceptable implication of governmental decisions to ration new cancer drugs to these participants.

These findings indicate that some clinicians may be unwilling to accept limits no matter how fair the process because the wider implications for the doctor-patient relationship or the privatisation of high-cost drug treatment are seen to be undermining the public healthcare system and thus unacceptable. The findings of Owen-Smith, Coast and Donovan [14] from a study of patient views on rationing decision underscore how notions of substantive justice as well as procedural fairness are important to perceptions of legitimacy. Our study supports these findings. In addition, our findings suggest that second-order beliefs about the justifiability of the rationing decisions are important for accepting decision-making in concrete cases. The implication may be that “trying to focus solely on process as a means to side-stepping potential moral disagreement” [37] is not an easy task, because the notions of fairness, reasonableness and transparency are not neutral terms and substantive values are indispensable for perceptions of just allocations. A broader concept of legitimacy than offered by frameworks such as Accountability for reasonableness seems to be called for.

### Limitations and further research

A potential limitation of this study is its generalisability. Each healthcare system is unique and the findings from our study of the Norwegian system may not be easily transferable to other contexts. Each local context shapes specific experiences and views. However, financial challenges are similar across political systems. The themes reported here are likely to be relevant to other settings as the difficult allocation decisions that follow from high-cost cancer treatments are present in health systems worldwide.

The sample was small and we do not know to which extent the sample resembles the population of interest. The participants in this study were specialists treating breast and prostate cancer, the most common cancers in Norwegian women and men, respectively. We do not know how oncologists in other sub-fields experience national rationing decisions.

Also, a specific challenge concerning expert interviewing is the asymmetric knowledge in favour of the participant [38]. The participants in this study were highly specialised experts who had considerable experience with rationing decisions. The participants were not necessarily objective and may have been tempted to signal “political” messages. This bias may have influenced the interview process.

Another limitation follows from the fact that this study analysed perceptions, not actual practice. While the participants were selected strategically because they had substantial knowledge of the subject matter, we cannot rule out the possibility that the participants’ perceptions about Norwegian priority setting criteria and processes may have been based on misunderstandings or false beliefs about facts.

Our hope is that the lessons from this study will be important to others who seek to develop explicit priority setting processes. Future research in this area should focus on perceptions of legitimacy of clinicians with experience from other areas than cancer treatment. Also, it would be fruitful to examine perceptions of legitimacy from the perspective of other stakeholder groups, such as patients.

## Conclusion

As governments are increasingly deciding not to fund promising high-cost drugs because effectiveness is low or uncertain, it is important that clinicians’ views about such rationing decisions are evaluated. This study offers insights into the under-researched area of how oncologists experience explicit rationing and their perceptions of legitimate limit-setting. Its intended contribution is to broaden our understanding regarding experiences of rationing at the micro-level and provide information for policy-makers interested in ensuring legitimacy in rationing scarce healthcare resources. Our findings suggest that perceptions of legitimacy may not only be formed by procedural features but also by substantive values that are realised and second-order beliefs about the justifiability of rationing new technology. These findings point at a need for a more sophisticated theoretical framework than is commonly used to assess the legitimacy of priority-setting and rationing.

## Abbreviations

CEO: Chief Executive Officer
QALY: Quality Adjusted Life Year
